# An experience of community mental health program in rural areas of Jharkhand

**DOI:** 10.4103/0972-6748.57860

**Published:** 2009

**Authors:** Shantna Kumari, S. N. Mishra, S. Chaudhury, Amool R. Singh, A. N. Verma, Sangeeta Kumari

**Affiliations:** Department of PSW, RINPAS, Hazaribagh, India; 1 Sociologist, NTPC, Rehabilitation Unit, Hazaribagh, India; 2 Department of Psychiatry, RINPAS, Delhi, India; 3 Department of Clinical Psychology, RINPAS, Delhi, India; 4 Department of Psychiatric Social Work, RINPAS, Delhi, India; 5 Psychiatric Social Worker, IHBAS, Delhi, India

**Keywords:** Community-based rehabilitation, Mental illness, Prevalence, Rural community

## Abstract

**Background:**

In the present era, mental disability is a major public health problem in the society. Many of the mental disabilities are correctable if detected early.

**Objectives:**

To assess the prevalence and pattern of mental disability.

**Materials and Methods:**

Community-based cross-sectional study. Patients of all age groups in the age range of 0-60 years were randomly selected from 10 blocks of 2 districts, viz., Ranchi and Hazaribagh. Thirty villages from each block were taken for the study. The study was conducted by making house-to-house visits, interviewing and examining all the individuals in the families selected using pre-tested questionnaire. Statistical Analysis: It was done by the proportions.

**Results and Conclusion:**

The prevalence of mental disability was found higher among males (67.9%) than among females (32.1%). The prevalence rate was higher among the productive groups and among individuals with low socioeconomic status. There is scope of community-based rehabilitation of the mentally disabled.

Mental disorders are prevalent in people of all regions, countries and societies. They affect men and women at all stages of life. Contrary to popular belief, the poor are more likely to suffer from mental and behavioral disorders (The World Health Report, 2001) and are more likely to suffer tragic outcomes as a result of their illness. The National Mental Health Program was developed in India to address the problem of mental illnesses, especially in rural areas. However, it has come under some criticism as it has laid emphasis on identifying and treating severe mental disorders such as psychosis, while not addressing common mental disorders [CMDs], which are equally disabling. CMDs, which are neurotic disorders presenting with anxiety and depressive symptoms, are widespread and are known to cause significant disability worldwide. In India, prevalence rates of CMD range from 2% to 57% (Patel, 1999). Majority of patients with CMDs present at primary care centers but end up receiving symptomatic treatments like painkillers and vitamins because their disorders are not recognized by primary care physicians as being mental illnesses. CMDs in such patients lead to chronic disability and progress in severity, making ultimate treatment more difficult (Issac *et al*., 2005). World Health Organization estimates that 10% of the world’s population has mental disabilities, and 1% suffers from severe incapacitating mental disorders. The disability-adjusted life year (DALY) loss due to neuropsychiatric disorders is much higher than that due to diarrhea, malaria, worm infestations and tuberculosis if taken individually. According to estimates, DALYs lost due to mental disorders are expected to represent 15% of the global burden of diseases by 2020.

During the last two decades, many epidemiological studies conducted in India show that the prevalence of major psychiatric disorders is about the same all over the world. The prevalences reported from these studies range from 18 to 207 per 1000 of the population, with a median of 65.4 per 1000; and at any given time, about 2% to 3% of the population suffer from seriously incapacitating mental disorders or epilepsy. A meta-analysis revealed that the prevalence of psychiatric disorders was around 5.8% (Reddy *et al*., 1998). Most of these patients live in rural areas, remote from any modern mental health facilities. A large number of adult patients (10.4%-53%) coming to the general OPD are diagnosed as being mentally ill. However, these patients are usually missed because either the medical officer or the general practitioner at the primary health care unit does not ask detailed mental health history. Due to the under-diagnosis of these patients, unnecessary investigations and treatments are offered, which put a heavy financial burden on the patients.

The Mental Health Act 1987 provides safeguards against stigmatization of patients suffering from mental illness. Community care of the chronic mentally ill has always been prevalent in India, largely due to family involvement and unavailability of institutions. In the 80s, a few mental health clinics became operational in some parts of the country. The Schizophrenia Research Foundation (SCARF), an NGO in Chennai, had established a community clinic in 1989 in Thiruporur, which was functional till 1999. Community mental health rehabilitation programs are carried out in a rural area in Jharkhand by the Nab Bharat Jagriti Kendra (NBJK), a nongovernmental, nonprofit organization working for people with physical and mental disabilities. The community mental health project is funded by Action Aid, India, and is carried out in Ranchi and Hazaribagh districts of Jharkhand. Covering 30 villages from 10 blocks, this area has a total population of 433,657 persons, most of them below the poverty line. One primary health center (PHC) and a few sub-centers cater to the health needs of the population. In these rural communities, faith healing and traditional medicines for mental illnesses are quiet popular and these traditional healers often are the first point of contact. The present study was conducted to understand the prevalence rate of mental disability and for developing community-based rehabilitation programs for the mentally ill.

## MATERIALS AND METHODS

This was a community-based cross-sectional study carried out over a period from January 2005 to December 2005. This study was conducted at the rural field of a nongovernmental organization Nab Bharat Jagriti Kendra, which covers a population of 1,332,739 spread over 10 blocks in the state of Jharkhand, India. A favorable sex ratio (Male: Female – 1000: 941) and literacy rate 54.1% in males and 39.45% were the striking features of this area. Mostly tribal people live in these areas. Two districts Ranchi and Hazaribagh and 5 blocks from each of these districts were chosen for the study. The sample size was estimated to be 433,657 persons. The study was conducted by making house-to-house visits and interviewing all the individuals in the families selected using a pre-tested questionnaire. Mental disability was assessed by ‘disability evaluation and assessment scale’ (IDEAS), a scale for measuring and quantifying disability in mental disorders, developed by the Rehabilitation Specialty Section of Indian Psychiatric Society (Ministry of Social Justice and Empowerment, Govt. of India, 2002). Disability in children below the age of 5 years was assessed based on a questionnaire designed on the lines of questionnaire taken from Action Aid India. Action Aid India instrument is used for the assessment of mental disability of a child. Children were examined and developmental delay in responding to the name or voice, smile, communication; and learning difficulties were noted down (Thomas *et al*., 2005). The data collected was tabulated by using proportions. Findings were described in terms of percentages. After knowing the status of mental illness, community-based rehabilitation program as under was planned and implemented.

### The mental health program

The primary objective was to operate a community-based mental health program in the defined catchment areas. The other program components included capacity-building program to detect mental disorders and make referrals for mental disorders, as well as linkages from government mental hospitals; awareness programs; services delivery; and rehabilitation programs.

### Capacity-building program

The community-based rehabilitation workers (CBRWs) were lay volunteers identified from the community with the help of *Gram Sabha*. The training consisted of 10 sessions each for the 10 groups of CBRWs. Other groups like Person with disability (PWD) self-help group (18), *panchayat*-level PWD communities (20), block-level PWD communities (2) and district-level PWD community (1) were formed and given training regarding mental illness and mental disabilities. They helped in identifying the case, implementation of simple intervention strategies, working closely with families of the mentally ill and making appropriate referrals. Manual and audiovisual training materials were used for the sessions.

### Awareness building

The awareness-building program included community sensitization camps, street plays, wall writing, pamphlets, posters, letters and booklet distribution containing all information related to, and meeting the needs of, disabled people in the catchment area of study.

### Sensitization and workshops

Several sensitization programs and workshops for key players, i.e., ANM (Auxillary Nurse Midwife), media, block administration BDOs (Block Development Officer), MOs (Medical Officer), CDPOs (Child Development Project Officer), other NGOs, school teachers, physicians and other medical officers, parents, village leaders, were conducted during the Mental Health Program.

### Mental health services

The identified patients and disabled were linked to the Ranchi Institute of Neuropsychiatry and Allied Sciences (RINPAS), Ranchi. They visited the patients with the help of CBRWs, and further checkup was done by psychiatrists and psychiatric social workers and the progress was reviewed monthly. A similar procedure was followed in camps held in villages which were not accessible to the clinic staff. Some psycho-social intervention included support to the patient, educating families on mental illness, management of behavioral problems, ensuring drug compliance, training patients in self care and daily living, job placements and helping them initiate small businesses as a measure of rehabilitation. The emphasis was on utilizing local resources and mobilizing local support. Over a period of 2 years, 2003-20053026 patients suffering from mental illness were registered and offered treatment; and by the end of the programs, 2112 patients were on medication, 585 were stable and 329 were self employed (rehabilitated). When we made our exit from the project area in 2005, we were quite satisfied with the pace of the activities we had initiated. Our major successes were as follows: Due to the mental health facilities in the region, there are now 3 mental hospitals run by government and a private body. RINPAS also has done camps in the remote areas and given free treatment and medication. This will help the patients to continue medication and come for follow-up.

## RESULTS

The prevalence of disability was the highest in the age groups 30-34 years and 20-24 years, which was significantly higher than the prevalence in other age groups. Among 1432 patients of psychosis, 269 had depression and anxiety, 503 had mental retardation, 358 had epilepsy and 464 had substance abuse or dependence. The total number of disabled individuals was 3026, among whom 1855 were males and 823 were females. The difference in prevalence of disability seen in males (67.9%) and females (32.1%) was not statistically significant. The overall prevalence of mental disability was 1% to 2% [Table 1].

**Table 1:** Prevalence and disability according to different diagnostic groups

Block	Population surveyed	Psychosis	Anxiety and depression	Mental retardation	Epilepsy	Substance dependence	Total	%
Muruhu	14537	218	20	68	20	40	366	2.518
Khunti	19512	145	35	70	53	75	378	1.937
Ormanjhi	32948	184	70	46	34	98	432	1.311
Angara	34042	90	15	22	45	122	294	0.864
Kanke	53775	124	17	64	65	16	286	0.532
Chauparan	75260	185	30	69	16	29	329	0.437
Churchu	40734	128	32	22	19	13	214	0.525
Ichak	52098	70	14	25	21	21	151	0.290
Katkamsandi	29551	67	12	53	61	19	212	0.717
Sadar	81200	221	24	64	24	31	364	0.448
Total	433657	1432	269	503	358	464	3026	-

The prevalence of disability was the lowest in the high socioeconomic group; except in case of mental retardation, which was higher in the high socioeconomic group. More males than females had psychosis and substance-dependence-related problems, whereas more females than males had problems of depression and anxiety [Tables 2 and 3].

**Table 2:** Prevalence and disability according to sex

Illness	Male	Female	Total
Psychosis	933	499	1432
Depression and anxieties	116	153	269
Mental retardation	362	141	503
Epilepsy	221	137	358
Substance dependence	423	41	464

**Table 3:** Distribution of disability according to age

Age	Psychosis	Anxiety and depression	Mental retardation	Epilepsy	Substance dependence	Total
0-4	0	0	220	54	0	274
005-9	0	1	135	15	0	151
10-014	0	6	67	21	0	94
15-19	16	19	47	42	3	127
20-24	367	36	9	36	104	552
25-29	217	30	6	28	112	393
30-34	323	63	7	112	174	679
35-44	237	36	2	13	32	320
45-59	103	24	7	20	17	171
60>	169	54	3	17	22	265
Total	1432	269	503	358	464	3026

## DISCUSSION

Well-documented studies to determine the prevalence and pattern of mental disorders are few. There are no community-based studies using IDEAS for assessment of disability, but there are some hospital-based studies among patients with mental illness and mental disability using IDEAS. This instrument was used for mental illnesses that included schizophrenia, bipolar affective disorders, anxiety disorders, depression, obsessive-compulsive disorders, dementia, behavioral disorders due to the intake of alcohol (Chaudhury *et al*., 2006; Mohan *et al*., 2005). The field workers involved in data collection could not detect mild degrees of disability because of their limited knowledge and lack of training. The present study showed a higher prevalence of mental disability in general (Chaudhury *et al*., 2006). The prevalence was more common among the productive age group. This has been attributed to higher prevalence due to socioeconomic conditions, marital and familial problems and substance abuse. Higher prevalence of mental disabilities in males is probably due to nonrespondent females. Moreover, family members often hide female patients with mental illness due to stigma. The prevalence of mental disability was the lowest among the group of persons with high socioeconomic status. In this area, the disabled were educated up to the primary level. About a third of the 7 to 8 million Indians who suffer from psychosis will be severely disabled and will require intense rehabilitation inputs (Census of India, 2001). The moderately disabled also require intervention, largely in relation to work and employment. After knowing the status of mental illness, community-based rehabilitation program was planned and implemented.

Jharkhand is rich in local resources like agriculture, forest and minerals, and so patients who improved with treatment and engaged in field or local business tended to drop out of follow up. But dropping out of psychiatric care is not an uncommon phenomenon, especially in the case of chronic mental illness. Several studies, including our own, have addressed this issue, while some have reported more dropouts among patients whose condition improved and who were satisfied with the level of care (Rossi *et al*., 2002). The contradictory finding of persons dropping out because of lack of improvement was seen in other studies (Rossi *et al*., 2002). Our own follow-up study revealed that those patients who had remitted had dropped out (Thara *et al*., 1990). This program has succeeded to an extent in attaining the objectives of the National Mental Health Program. Minimum mental health care is made accessible, available and affordable to the underprivileged sections of the population. Community health workers can play an important role in disseminating correct information regarding these disorders to the community and in reducing stigma. There are usually many myths and misconceptions associated with severe mental disorders in rural areas, and these are very resistant to change. However, the cure of one such patient in a village is enough to change people’s attitudes.

### Limitations

A limitation of our study was that we could not interview nonrespondents because of their noncooperation or non-availability during our field visits; hence the entire population could not be covered.

## CONCLUSION

Common mental disorders form a large proportion of the total burden of mental illnesses and must be addressed in all mental health programs. Collaboration between the Departments of Psychiatry and Psychiatric Social Work and NGOs is useful in developing such programs at the primary care level. In our initiative, postgraduate students of both departments got an opportunity to view the entire spectrum of mental illnesses and study the social, economic and cultural factors that were intertwined with them.
